# Quantitative analysis of retinal vascular parameters changes in school-age children with refractive error using artificial intelligence

**DOI:** 10.3389/fmed.2024.1528772

**Published:** 2024-12-31

**Authors:** Linlin Liu, Lijie Zhong, Linggeng Zeng, Fang Liu, Xinghui Yu, Lianfeng Xie, Shuxiang Tan, Shuang Zhang, Yi-Ping Jiang

**Affiliations:** ^1^The Department of Ophthalmology of the First Affiliated Hospital, Gannan Medical University, Ganzhou, Jiangxi, China; ^2^Postgraduates at the First Clinical Medicine of Gannan Medical University, Ganzhou, Jiangxi, China

**Keywords:** artificial intelligence, quantitative analysis, ametropia, retinal vascular changes, school-age children

## Abstract

**Aim:**

To quantitatively analyze the relationship between spherical equivalent refraction (SER) and retinal vascular changes in school-age children with refractive error by applying fundus photography combined with artificial intelligence (AI) technology and explore the structural changes in retinal vasculature in these children.

**Methods:**

We conducted a retrospective case–control study, collecting data on 113 cases involving 226 eyes of schoolchildren aged 6–12 years who attended outpatient clinics in our hospital between October 2021 and May 2022. Based on the refractive spherical equivalent refraction, we categorized the participants into four groups: 66 eyes in the low myopia group, 60 eyes in the intermediate myopia group, 50 eyes in the high myopia group, and 50 eyes in the control group. All participants underwent a series of examinations, including naked-eye and best-corrected visual acuity, cycloplegic spherical equivalent refraction, intraocular pressure measurement, ocular axial measurement (AL), and color fundus photography. Using fundus photography, we quantitatively analyzed changes in the retinal vascular arteriovenous ratio (AVR), average curvature, and vascular density with AI technology. Data were analyzed using the χ^2^ test and one-way analysis of variance.

**Results:**

The AVR in the low myopia group, moderate myopia group, high myopia group, and control group were 0.80 ± 0.05, 0.80 ± 0.04, 0.76 ± 0.04, and 0.79 ± 0.04, respectively, and the vessel densities were 0.1024 ± 0.0076, 0.1024 ± 0.0074, 0.0880 ± 0.0126, and 0.1037 ± 0.0143, respectively The difference between the AVR and vascular density in the high myopia group was statistically significant compared to the other three groups (*p* < 0.05). Linear correlation analysis showed a strong negative correlation between the spherical equivalent refraction and the ocular axis (*r* = −0.874, *p* < 0001), a moderate positive correlation between the spherical equivalent refraction and the vascular density (*r* = 0.527, *p* < 0001), and a moderate negative correlation between the ocular axis and the vascular density (*r* = −0.452, *p* < 0001).

**Conclusion:**

Schoolchildren with high myopia showed a decreased AVR and decreased vascular density in the retinal vasculature. The AVR and vascular density may be early predictors of myopia progression.

## 1 Introduction

Myopia is a common ocular disease worldwide. Researchers have predicted that by 2050, 4.758 billion people will have myopia (49.8% of the world’s total population), and 938 million people will have high myopia (9.8% of the world’s total population). The rapid increase in the prevalence of myopia has become a global public health concern (1). High myopia is typically defined as a spherical refractive error of more than −6.00 DS or an ocular axis length more than 26 mm (2). Elongation of the AL and thinning of the retina, which often cause a series of degenerative changes in the fundus of pathological myopia, such as fundus tessellation (FT), temporal peripapillary chorioretinal atrophy, posterior scleral staphyloma, lacquer crack, myopic retinoschisis, and macular degeneration. These changes are among the most common causes of blindness in China (3).

In recent years, the application of artificial intelligence (AI) has shown great potential in the detection and diagnosis of early fundus lesions, not only extracting accurate data from fundus photography but also predicting the progression of the lesion (4), which has enabled clinical screening with its “low input and high output” (5). The application of AI technology for the prevention and control of myopia is one of the hotspots in ophthalmology. In order to investigate the relationship between the changes of retinal vascular parameters and different degrees of myopia, we conducted this study. In this study, we applied fundus photography and AI technology to quantitatively analyze the relationship between the spherical equivalent refraction and the ocular axis and retinal vascular alignment changes in school-age children with refractive error to investigate the changes in their retinal vascular structure and to provide theoretical data for the prevention and control of myopia. The results are as follows.

## 2 Materials and methods

### 2.1 Data

In this study, data on 113 participants involving 226 eyes of school children aged 6–12 years (118 male and 118 female) attending the outpatient clinic of the First Affiliated Hospital of Gannan Medical College from October 2021 to May 2022 were collected. We divided the participants into four groups based on their spherical equivalent (SE) values: 66 eyes in the low myopia group (−0.50D to −2.75D), 60 eyes in the moderate myopia group (−3.00D to −6.00D), 50 eyes in the high myopia group (below −6.00D), and 50 eyes in the control group (+0.25D to −0.25D). All the experimental groups included children diagnosed with refractive error and average-corrected visual acuity after cycloplegia. Emmetropia attending the same clinic were selected as the control group. The exclusion criteria included (1) amblyopia; according to the consensus of amblyopia diagnosis experts in 2011, best corrected visual acuity (BCVA) of both eyes lower than the corresponding age for amblyopia: <0.6 for 4–5 years old, <0.7 for 6–7 years old, and < 0.8 for more than 7 years old; (2) The anisometropia is more than 1.0 D between the two eyes; (3) refractive stromal opacity, which affected the imaging effect; (4) a history of systemic diseases and ophthalmic diseases; (5) a history of eye surgery and trauma; (6) difficulty with analyzing the quality of the collected image; and (7) inability to cooperate with the examination. The study was conducted in compliance with the Declaration of Helsinki and approved by the Hospital Ethics Committee.

### 2.2 Methodology

#### 2.2.1 Routine examination

Detailed and comprehensive ophthalmic examination was performed for all enrolled subjects, including un-aided eye visual acuity, BCVA, dilated pupil optometry, intraocular pressure (IOP), slit-lamp examination, fundus examination, ocular axial measurement, and color fundus photography. An ophthalmic optical biometrics instrument (IOL Master 500; Carl Zeiss Meditec, Jena, Germany) was used to measure the ocular axis. The ocular axis was measured continuously five times using the lens mode, and the average value was taken. A retinal camera (Canon CX-1; Canon Corp, Tokyo, Japan) was used for fundus color photography, and an AI system was used to quantitatively analyze fundus nerves and blood vessels.

#### 2.2.2 AI data acquisition

**Figure 1:**
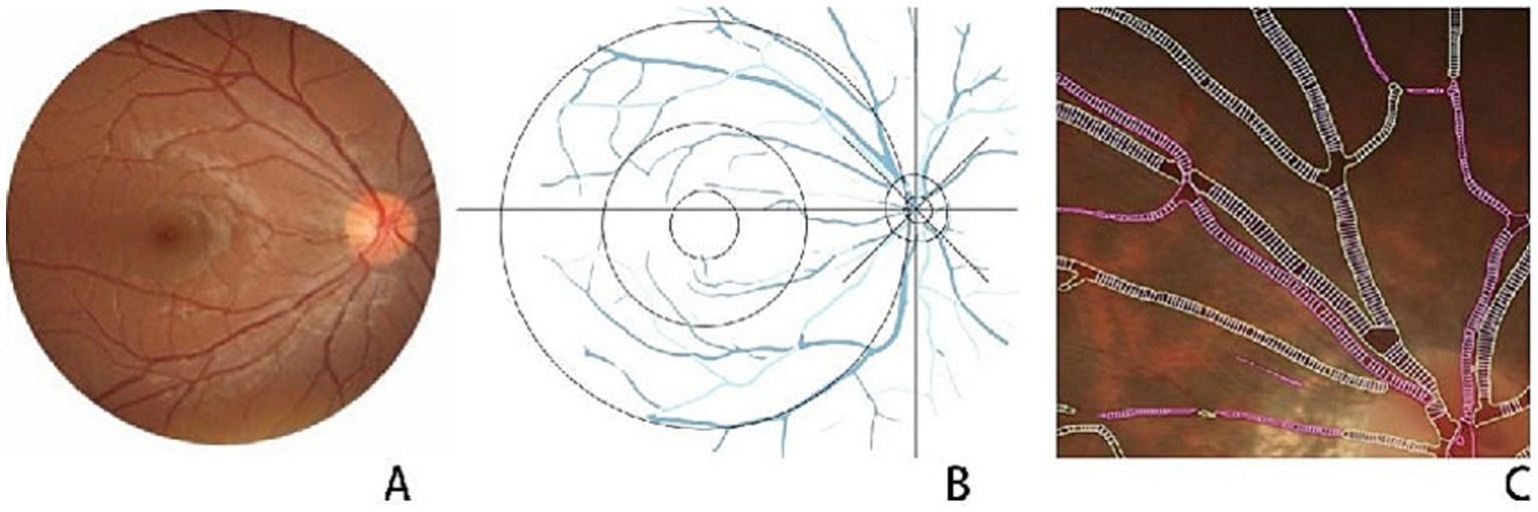
**(A)** Shows the original image of the fundus. **(B)** Artificial intelligence (AI) technology automatically extracted vital structures in the fundus, including blood vessels, optic disc, optic cup, macular center, and critical foci. We removed the undertones and plotted them in coordinates centered on the optic disc. **(C)** AI analyzed a localized magnified view of the retinal blood vessels; the red areas are arterial vessels and the green areas are venous vessels.

Data acquisition was performed using digitized color fundus photographs and a quantitative analysis system for fundus nerves and blood vessels (EVision AI) (6). Based on the bionic mechanism of human vision, EVision processed the fundus photographs using AI technologies such as computer vision and deep learning (7–9); it thoroughly combined the brightness, color, texture, morphology, topological features, and other detailed information expressed by the features on the image, and segmented the fundus structure on the image with fine features (10). Based on segmentation results, the features were quantified, and data such as the diameter and area of optic cup and optic disc, cup-to-disc ratio (C/D), vascular diameter, curvature, and AVR were measured to digitally represent the fundus image and fundus structure in an all-round way, which provides a more detailed understanding of fundus abnormalities and enables intelligent evaluation and detection of related fundus diseases (Figure 1).

#### 2.2.3 Statistical analysis

SPSS 20.0 software was used for statistical analysis, and the χ^2^ test was used to compare the two groups of count data; one-way analysis of variance (ANOVA) was used to compare the measured data between multiple groups, and *post hoc* multiple comparisons were performed using the least significant difference (LSD) method. Statistical significance was set at *p* < 0.05.

## 3 Results

### 3.1 Comparison of basic information

**Figure 2:**
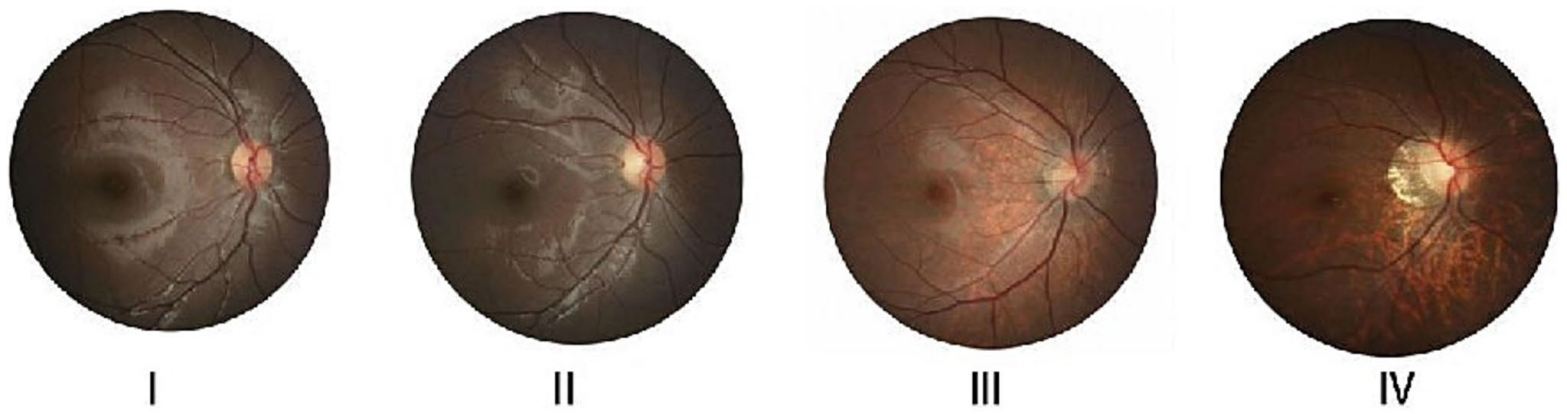
The typical fundus images of different refractive states. I, II, III, and IV represent the standard control, low myopia, moderate myopia, and high myopia groups, respectively.

Table 1 presents a comparison of age, gender, IOP, SE, and AL among the four groups of school-age children. The differences in age and gender between the four groups were not statistically significant (*p* > 0.05); two-by-two comparisons of SE and AL between the four groups showed that the differences were statistically significant (*p* < 0.05; Figure 2).

**Table 1:** Comparison of baseline data.

Characteristics	High myopia group (*n* = 50 eyes)	Moderate myopia group (*n* = 60 eyes)	Low myopia group (*n* = 66 eyes)	Normal control group (*n* = 50 eyes)	*χ* ^2^ */F*	*p*-value
Age (y)	10.12 ± 1.45	10.50 ± 2.09	10.08 ± 2.04	9.86 ± 1.83	1.114	0.344
Gender (M/F)	30/20	33/27	28/38	27/23	4.001	0.261
Mean intraocular Pressure (mmHg)	15.82 ± 2.96	16.44 ± 3.19	15.54 ± 2.84	16.74 ± 2.45	2.081	0.014
SE (DS)	−8.20 ± 1,96	−4.28 ± 0.79	−1.98 ± 0.67	0.46 ± 0.44	551.829	<0.001*
AL (mm)	26.75 ± 0.75	25.17 ± 0.67	24.39 ± 0.87	22.82 ± 1.01	133.012	<0.001*

*Indicates statistical differences between all four groups.

### 3.2 Quantitative analysis of fundus vasculature in patients with different refractive states

A statistically significant difference (*p* < 0.05) was observed in the AVR and vascular density between the highly myopic group and the other three groups as well as in the average curvature of blood vessels (*p* < 0.05) between the two groups in each of the four comparisons: the low myopia and the high myopia groups; the moderate myopia and the high myopia groups, the moderate myopia and the standard control groups; and the high myopia and the standard control groups. The two-by-two comparison of the mean vessel diameter of the groups indicated that the difference was statistically significant (*p* < 0.05) between the low myopia and the standard control groups; the moderate myopia and the high myopia groups; and the moderate myopia and the standard control groups (Table 2).

**Table 2:** Quantitative analysis of fundus vasculature in different refractive states.

	Arteriovenous ratio	Mean vascular curvature	Mean vascular diameter (μm)	Vascular density
Low Myopia Group	0.80 ± 0.05	0.00086 ± 0.00013	56.71 ± 2.32	0.1024 ± 0.0076
Moderate Myopia Group	0.80 ± 0.04	0.00084 ± 0.00013	56.25 ± 1.99	0.1024 ± 0.0074
High Myopia Group	0.76 ± 0.04	0.00075 ± 0.00008	57.28 ± 2.79	0.0880 ± 0.0126
Normal control group	0.79 ± 0.04	0.00090 ± 0.00017	57.67 ± 2.27	0.1037 ± 0.0143
*F*	9.280	12.125	3.925	25.463
*p*-value	0.000*	0.000#	0.009##	0.000*

Two-by-two comparison results show: *indicates a statistical difference between Groups 3 and 1,2,4; # indicates a statistical difference between groups 1–3, 2–3, 2–4, and 3–4; ## indicates a statistical difference between groups 1–4, 2–3, and 2–4.

### 3.3 Correlation analysis of retinal vascular parameters with SE and AL in patients with different refractive states

**Figure 3:**
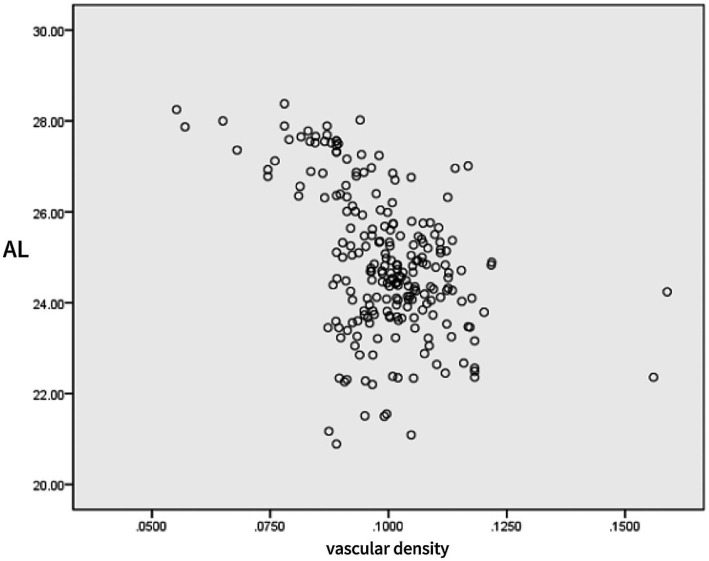
Scatter plot of eye axis and retinal vessel density.

**Figure 4:**
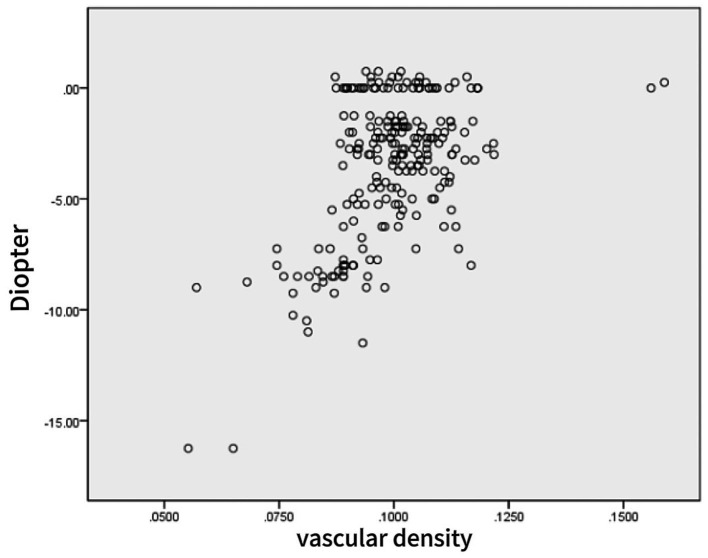
Scatter plot of spherical equivalent and retinal vessel density.

Linear correlation analysis showed a strong negative correlation between the SE and AL (*r* = −0.874, *p* < 0001), a moderate positive correlation between the SE and the vascular density (*r* = 0.527, *p* < 0001), and a moderate negative correlation between AL and the vascular density (*r* = −0.452, *p* < 0001) (Table 3 and Figures 3, 4).

**Table 3:** Correlation analysis of retinal vascular parameters with SE and AL.

	SE		AL	
	*r*	*p*	*r*	*p*
AL	−0.874	<0.001	1	–
SE	1	–	−0.874	<0.001
AVR	0.269	0.010	−0.282	<0.001
Mean vascular curvature	0.404	<0.001	−0.415	<0.001
Mean vascular diameter	0.098	0.144	−0.170	0.010
Vascular density	0.527	<0.001	−0.452	<0.001

## 4 Discussion

The results of this study showed that in school-age children with high myopia, the retinal vasculature had decreased AVR and vascular density; AVR and vascular density may serve as early predictors of myopia progression. We hypothesized that as the eye axis elongates, it physically stretches the retina, narrows the retinal vessels, lowers local oxygen consumption in the retina, and consequently causes more capillary loss and, ultimately, a decrease in retinal vascular density. AVR and vascular density are essential indicators of the retinal vascular structure, and their changes can reflect the physiological and pathological status of the eye. In the current study, we found a positive correlation between spherical equivalent and vascular density using correlation analysis, whereas we observed a negative correlation between ocular axis length and vascular density. This result confirms the close relationship between retinal vascular structure and myopia progression. As myopia deepens and the eye axis grows, the retinal vascular structure undergoes significant changes, which may substantially affect the progression of myopia and the development of complications.

Refractive errors are critical ocular problems that affect the visual development of school-age children and cover a wide range of types, including myopia, hyperopia, and astigmatism. These problems not only directly threaten children’s visual health but may lead to severe ocular diseases (11, 12). Particularly in cases of high myopia, as the ocular axis elongates further, various pathological changes can manifest in the fundus, such as optic disc tilt, fundus tessellation (FT), posterior scleral staphyloma, and macular degeneration. These conditions can result in severe visual impairment or blindness (3). Notably, Myopic Maculopathy (MM) has become a major cause of monocular blindness in East Asian countries (13). The prevalence of high myopia in Asian school-age children (6.7–21.6%) is much higher than that in non-Asian populations (2.0–2.3%), and the rising prevalence of high myopia will eventually lead to an increased prevalence of pathologic myopia (PM), which can cause irreversible vision loss (14). Studies have shown that an increased ocular axis length and spherical equivalent power are associated with the onset of visual impairment (15). Researchers have discovered that the ocular axis continues to elongate in adults with high myopia and that the risk factors for this growth are uncontrollable (16). Increasing age, elevated refractive error, and lengthening of the ocular axis in patients with high myopia are important risk factors for the progression of fundus lesions (17, 18).

In recent years, with the rapid development of AI technology, its application in the medical field has become increasingly widespread, especially in ophthalmology, providing new tools and methods for disease diagnosis and treatment (19–21). AI technology has led medicine into an era of precision medicine, realizing the transformation from qualitative to quantitative image analysis (22, 23). Early screening and determination of some morphological indicators of the fundus, using AI, can help accurately understand the degree of progression of myopic fundus changes, predict the pattern of myopic development, and enable timely interventions to prevent the development of myopia and its complications, which can help minimize the occurrence of irreversible visual impairment (11). Using AI technology to predict the progression of myopia in school-age children and design precise diagnostic and therapeutic protocols for them would potentially reduce the probability of their progression to high myopia or even PM (5, 24). As an essential part of the eye, the retinal vasculature, with changes in its morphology and lineage, can reflect physiological and pathological states within the eye (25). AI technology has a unique advantage in quantitatively analyzing the retinal vascular travel diameter. Through deep learning algorithms, the morphology and travel diameter information of retinal blood vessels can be automatically recognized and extracted, significantly improving the accuracy and efficiency of the analysis (26). Therefore, quantitative analysis of the retinal vascular travel diameter in school-age children with refractive error can help gain insights into their ocular pathology and provide strong support for diagnosing and treating the disease.

Researchers have discovered that certain morphological features of the fundus can indicate the progression of fundus lesions. For example, deepening myopia causes changes in the shape of the optic disc (27, 28), and increasing myopia may cause tilting of the optic disc, rotation, enlargement of the area, reduction of thickness, ratio of the area to peripapillary atrophy (PPA), and alterations in retinal thickness (29–32). An adequate blood flow is the basis for maintaining tissue homeostasis (33). A decrease in arteriovenous vasculature density may cause pathological changes in the fundus. Changes in the arteriovenous vasculature play a crucial role in the development of myopic maculopathy (34), and future studies on arteriovenous vasculature and fundus morphology in the eyes of patients with myopia are essential for preserving myopic eye health (35).

Several studies have shown that an increase in refractive error and axial length in patients with myopia causes changes in fundus morphology, and that there is a specific connection between the changes in different anatomical landmarks of each fundus; researchers are continually searching for indicators related to fundus morphologic markers (36, 37). More accurate quantitative detection of vascular alignment in the fundus of patients with refractive errors is necessary. In previous studies by Azemin et al., changes in vascular density across various refractive states were compared using fundus photography and vascular density was assessed using fractal dimension (FD), and the results showed that retinal vascular density was lower in myopic than in orthokeratology populations (1, 38). Although some researchers have used ImageJ image analysis software to correlate optic disc structure and fundus morphological markers in patients with high myopia (39) and have obtained data on fundus structure for optic disc structure analysis, ImageJ is a manual annotation software that is inefficient and has poor precision. We performed this preliminary study because of the paucity of studies on quantitative analysis of fundus structures by AI. In this study, we grouped 6–12-year-olds with refractive errors according to different refractive states, and measured some of the anatomical markers of the fundus extracted from fundus photography through automated quantitative measurement of AI software, accurately measured the values of the fundus AVR and vascular density-related indices, and analyzed the correlation between these indices and refractive errors. Our results provide an essential basis for further understanding the mechanisms of progression of fundus lesions in children with refractive errors and provide scientific support for developing more effective prevention and control strategies for myopia.

This study has the following limitations: (1) The quality of data and the accuracy of the labeling have a considerable impact on the model’s performance, and the comparison and validation between different algorithms and models is also an issue of concern, (2) The sample size is too small, and (3) As a cross-sectional, retrospective study, the sample required follow-up, which limited the depth and persuasiveness of the results. We plan to increase future follow-ups to observe long-term changes in the fundus structure.

In summary, the present study quantitatively analyzed the vascular structure of the fundus in school-age children and found that the retinal vessels of children with high myopia had decreased AVR and vascular density, which may be closely related to myopia progression. Although this study has some limitations, it provides new ideas and methods for early intervention in myopia, and essential references and insights for future research. In the future, we will continue to perform in-depth research in this field to make more impactful contributions toward improving children’s visual health.

## Data Availability

The original contributions presented in the study are included in the article/supplementary material, further inquiries can be directed to the corresponding author.
